# Evolved hexose transporter enhances xylose uptake and glucose/xylose co-utilization in *Saccharomyces cerevisiae*

**DOI:** 10.1038/srep19512

**Published:** 2016-01-19

**Authors:** Amanda Reider Apel, Mario Ouellet, Heather Szmidt-Middleton, Jay D. Keasling, Aindrila Mukhopadhyay

**Affiliations:** 1 Joint BioEnergy Institute, 5885 Hollis St, Emeryville, CA 94608, USA; 2 Biological Systems and Engineering Division, Lawrence Berkeley National Laboratory, Berkeley, CA 94720, USA; 3 Department of Bioengineering, University of California, Berkeley, CA 94720, USA; 4 Department of Chemical & Biomolecular Engineering, University of California, Berkeley, CA 94720, USA

## Abstract

Enhancing xylose utilization has been a major focus in *Saccharomyces cerevisiae* strain-engineering efforts. The incentive for these studies arises from the need to use all sugars in the typical carbon mixtures that comprise standard renewable plant-biomass-based carbon sources. While major advances have been made in developing utilization pathways, the efficient import of five carbon sugars into the cell remains an important bottleneck in this endeavor. Here we use an engineered *S. cerevisiae* BY4742 strain, containing an established heterologous xylose utilization pathway, and imposed a laboratory evolution regime with xylose as the sole carbon source. We obtained several evolved strains with improved growth phenotypes and evaluated the best candidate using genome resequencing. We observed remarkably few single nucleotide polymorphisms in the evolved strain, among which we confirmed a single amino acid change in the hexose transporter *HXT7* coding sequence to be responsible for the evolved phenotype. The mutant *HXT7(F79S)* shows improved xylose uptake rates (Vmax = 186.4 ± 20.1 nmol•min^−1^•mg^−1^) that allows the *S. cerevisiae* strain to show significant growth with xylose as the sole carbon source, as well as partial co-utilization of glucose and xylose in a mixed sugar cultivation.

In order to cost-effectively produce biofuels from renewable plant biomass, all sugars, including all pentose and hexose sugars present in the raw lignocellulosic starting material, must be converted efficiently into the final products^1^. The yeast, *S. cerevisiae*, is an excellent host-microbe for a range of industrial applications, from chemical and commodity production, to biofuel synthesis^2^,^3^,^4^. However, *S. cerevisiae* does not readily uptake and use pentose sugars. This includes xylose, the most abundant pentose, and the second most abundant sugar next to glucose, found in biomass^5^. While native xylose-utilizing organisms exist, they largely lack well-developed genetic tools for host engineering or exhibit low product and inhibitor tolerances. Therefore, it is important to develop *S. cerevisiae* host platforms with more efficient xylose utilization.

Generating a yeast strain that utilizes xylose, especially in a glucose/ xylose mix has been an object of extensive research for several decades^6^. Great success has been achieved in boosting the native yeast utilization capability. Two approaches are now used routinely to provide for xylose utilization: overexpression of a heterologous xylose isomerase (XI)^7^,^8^,^9^,^10^,^11^, and overexpression of the native or heterologous xylose reductase (XR) and xylitol dehydrogenase (XDH)^12^,^13^. Both pathways result in the transformation of xylose to xylulose, and benefit from additional overexpression of xylulokinase (XKS) to shunt the carbon into pentose-phosphate pathway (PPP)^14^,^15^. Further, overexpression of genes encoding enzymes in the pentose-phosphate pathway, such as the transaldolase (*TAL1)* and the transketolase (*TKL1)*, leads to additional improvements in xylose assimilation rates^7^,^16^,^17^,^18^. Recently, it has also been shown that xylose utilization can be achieved via replacement of the native *S. cerevisiae* xylose utilization and PPP genes with those from the xylose-utilizing yeast *Scheffersomyces stipitis*^19^.

The improvements in intracellular xylose consumption have led to a bottleneck in xylose uptake^20^. To date there has been no discovery of a sugar transporter that, in *S. cerevisiae*, allows for xylose uptake comparable to glucose uptake. *S. cerevisiae* has numerous monosaccharide transporters (*HXT1-17* and *GAL2)*, but all of them have greater specificity for hexose sugars. While a few of these (*HXT1, 2, 4, 5, 7* and *GAL2)* can import xylose, they display rates of uptake so low that they cannot support growth on xylose^6^,^21^,^22^,^23^,^24^,^25^. Further, xylose uptake in these native transporters is repressed in the presence of glucose, limiting the use of these transporters in mixed sugar sources^26^,^27^.

Several strategies have been employed to tackle the issues with xylose transport. Much work has been devoted to bioprospecting and characterizing heterologous xylose-transporters in *S. cerevisiae*, resulting in the identification of several membrane proteins that can transport xylose^22^,^28^,^29^,^30^,^31^,^32^,^33^. These studies have shown that increasing xylose transport does increase utilization and final product formation, proving that xylose import is the limiting factor in utilization. However, these transporters have had limited efficacy either due to reduced growth rates, problems with substrate affinities, non-optimal transport rates, or substrate inhibition.

Recently, a few studies have attempted to improve transport by engineering native transporters with encouraging results. Using a combination of bioinformatics, and mutagenesis, Young and colleagues, identified a xylose transport sequence motif, and were able to produce a mutant *HXT7* strain that grew on xylose, but not glucose^34^. Although this strain still showed glucose inhibition, another group was able to bypass this problem using growth to screen for mutants with glucose insensitivity^35^. This latter approach resulted in the discovery of Gal2 and Hxt7 variants that bypass glucose inhibition. Unfortunately, the modifications that eliminated glucose repression also resulted in diminished uptake rates (Vmax). Additionally, although the transporter is overexpressed, the resulting growth on xylose was modest in both these studies and would benefit from further optimization.

In the present study, we used an evolutionary engineering approach to address the problem of xylose import. Starting with a *S. cerevisiae* strain that has been engineered to enhance intracellular xylose consumption, we report the discovery of a mutation in *HXT7* that shows improved xylose uptake rates, and allows *S. cerevisiae* to show significant growth with xylose as the sole carbon source. This mutation, F79S, is predicted to lie within the first transmembrane region of the transporter and enables partial co-utilization in a glucose/ xylose mix.

## Results

### Evolution of a xylose utilizing strain

**Figure 1:**
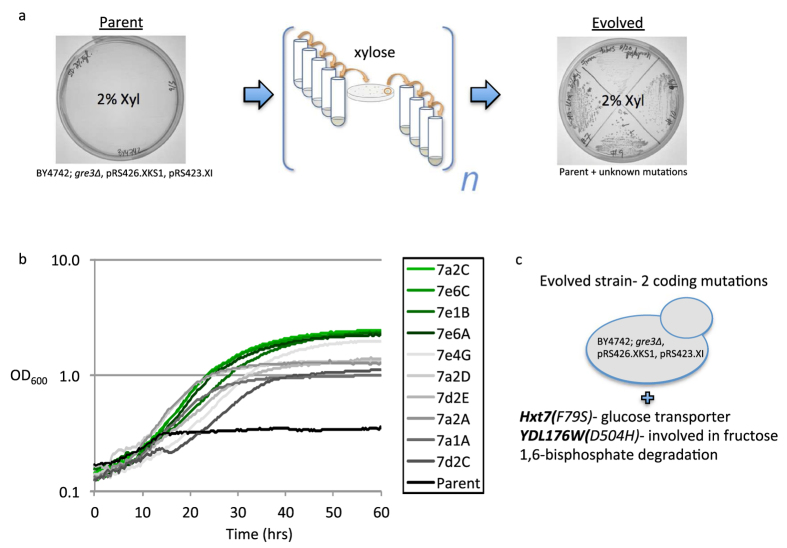
Laboratory evolution of a xylose utilizing strain. (**a**) Growth of the parent strain (JBEI_ScMO001) and four representative xylose-evolved strains. Genotype of each strain listed below panel showing growth. (**b**) Growth curves of the parent strain and fastest growing evolved strains. OD_600_ was measured every 18 minutes for 72 hours. Y- axis is shown in log base 10 scale. The strains in panels (**a,b**) were grown on or in SD, 2% xylose medium at 30 °C. (**c**) Genomic sequencing of the evolved strain (JBEI_ScMO002) disclosed the presence of three SNPs, two in coding regions.

Since xylose import into the cell is a limiting factor in *S. cerevisiae* growth and utilization of xylose, we hypothesized that we could select for increased xylose uptake by subjecting a *S. cerevisiae* strain engineered with an improved cytosolic xylose metabolic pathway to evolution in xylose medium (i.e. xylose as the sole carbon source). JBEI_ScMO001, a BY4742 strain deleted for the XR, *gre3*, and overexpressing the *Piromyces sp*. XI*, pspXI*, and *XKS1*, was sub-cultured in synthetic defined (SD), 2% xylose medium. After several rounds of sub-culturing, the culture was plated onto solid xylose medium and the fastest growing colonies were selected (Fig. 1a). The clones were assayed for growth and xylose consumption and the best performing strains were further evolved in SD, 2% xylose medium. This process was repeated until strains were obtained where growth could be seen in one day. The doubling time of the fastest-growing strains in xylose were reduced to approximately nine hours, down from an initial doubling time of over 150 hours for the unevolved strain (Fig. 1b). Colonies that showed improved xylose utilization were confirmed to be *S. cerevisiae* via 16S sequencing. Other eukaryotic contaminants, such as *Aureobasidium pullulans* were also detected, but not selected for sequencing.

The fastest-growing, xylose-utilizing *S. cerevisiae* strain, 7a2c (JBEI_ScMO002), was selected and analyzed for mutations by whole-genome sequencing. Sequencing revealed single nucleotide polymorphisms (SNPs) in three genes, including a mutation in the hexose transporter, *HXT7*. Additional mutations were found in *YDL176W*, a gene predicted to be involved in fructose-1,6-bisphosphatase degradation, as well as in an intergenic region on the left telomere of chromosome eight (Fig. 1c). Because the mutation in chromosome eight was in a heterochromatic region it was not pursued further.

### *HXT7(F79S)* confers growth in xylose medium

**Figure 2:**
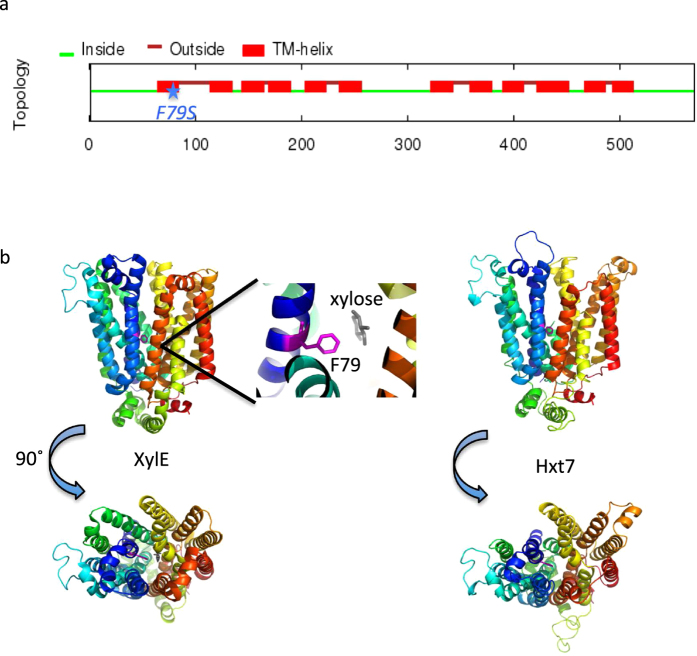
Structural models of Hxt7. (**a**) Topology prediction of Hxt7 was generated using Spoctopus (36). Red boxes indicate transmembrane regions (12 in total), green lines denote cytosolic regions, and red lines represent extracellular domains. The Hxt7(F79S) mutation is marked by a blue star, and is predicted to reside in the middle of transmembrane helix one. (**b**) Homology model of the Hxt7 structure. The theoretical structure of Hxt7 was generated from the structure of *E. coli* XylE with bound xylose (PDB: 4GBY) Left: Side and top view of XylE structure; zoom inset of Phe (pink) homologous to the mutated F79 of Hxt7 in close proximity to xylose (gray). Right: Side and top view of the predicted structure of Hxt7. Each peptide is colored from N (blue) to C-terminus (red).

Since Hxt7 is a known hexose transporter that can also transport pentose sugars with low affinity, the *HXT7(F79S)* mutation was our most likely candidate for conferring growth in xylose. Like other Hxt proteins, SPOCTOPUS software^36^ predicted Hxt7 to be a 12-pass transmembrane protein with the F79S mutation located in the first predicted membrane helix (Fig. 2a). Since there is no solved structure for any of the Hxt transport proteins, Phyre software^37^ was used to predict the structure of Hxt7 based upon its closest homolog with a solved structure, the bacterial XylE (Fig. 2b). The model predicted that residue F79 resides in the middle of helix one, facing internally towards the central pore. The recently solved structure of XylE has the added benefit that it was crystalized in complex with xylose and glucose, conveying fundamental information about substrate binding^38^. Intriguingly, Hxt7 F79 lies in close proximity to the bound-xylose in the pore of the XylE structure, and therefore suggests that the residue is poised to affect xylose binding and transport (Fig. 2b).

**Figure 3:**
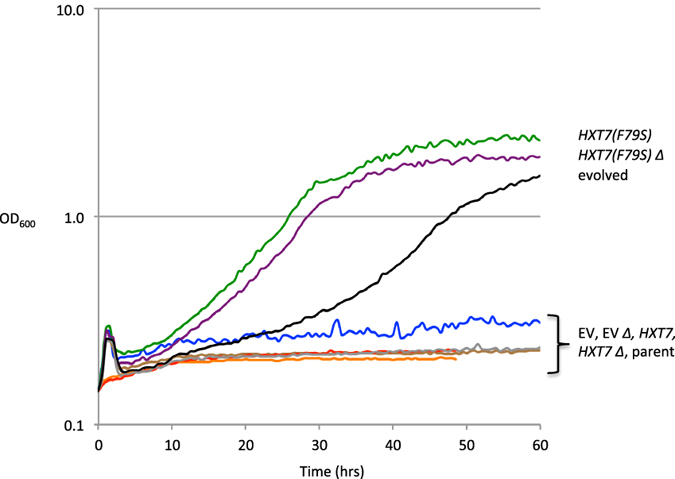
*HXT7(F79S)* is responsible for growth on xylose. Low-copy, *HXT7, HXT7(F79S)* or empty vector (EV) were expressed in strains wild-type (JBEI-9005 EV; JBEI-9006 *HXT7(F79S)*; JBEI-9007 *HXT7*) or deleted (∆) for *hxt7* (JBEI-9008 EV; JBEI-9009 *HXT7(F79S)*; JBEI-9010 *HXT7*). The strains were grown in SD, 2% xylose medium at 30 °C, and the OD_600_ was measured every 15 minutes for 60 hours. The experiment was conducted in quintuplicate, and the representative curves are shown for clarity. Replicates have no more than 10% error. Y- axis is shown in log base 10 scale.

To test if the *HXT7(F79S)* mutation was indeed responsible for the improved growth in xylose, we individually cloned each mutated gene, *HXT7(F79S)* or *YDL176W(D504W)*, into single-copy plasmids, under their native promoters and terminators, and transformed the resulting plasmids into the *gre3*∆ strain overexpressing *pspXI*, *XKS1, and TAL1*. The plasmids were also transformed into a strain that contained additional deletions in the genes of interests (*hxt7*; *ydl176w*). The transformants were examined for growth in SD, 2% xylose medium. Both the *gre3*∆ and *gre3*∆ *hxt7∆* strains expressing *HXT7(F79S)* grew in xylose medium, reaching a maximum optical density (OD_600_) of between 2.0–2.4 after 40 hours. The two strains transformed with empty vector plasmids showed no growth after 60 hours (Fig. 3). To eliminate the possibility that an extra copy of *HXT7* permits growth in xylose medium, wild-type *HXT7* was also expressed in the *gre3∆* and *gre3∆ hxt7∆* strains and tested for growth. However, these strains did not grow in the xylose medium (Fig. 3), confirming that the xylose growth is specific to the *HXT7(F79S)* mutation. Of note, the evolved strain showed a longer lag time in this assay then in the assay performed in Fig. 1b. This is due to the glucose pre-culturing conditions needed for the growth of the controls. When the evolved strain is pre-cultured in a glucose medium prior to culturing in a xylose medium, we observe an approximate 12-hour increase in lag time (Fig. S1; evolved gx) relative to the same strain pre-grown in xylose medium (Fig. S1; evolved xx). This further suggests that Hxt7 is the causal mutation, as its expression is known to be repressed by glucose^39^.

*YDL176W(D504H)* did not contribute significantly to the growth of the evolved strain in xylose. Strains expressing the *YDL176W(D504H)* alone showed no growth in SD, 2% xylose medium, while strains expressing *YDL176W(D504H)* along with a wild-type genomic copy only showed marginal growth to OD_600_ 0.6 after 60 hours (Fig. S2).

### *HXT7(F79S)* allows for increased xylose consumption and partial co-utilization in a mixed-sugar source

**Figure 4:**
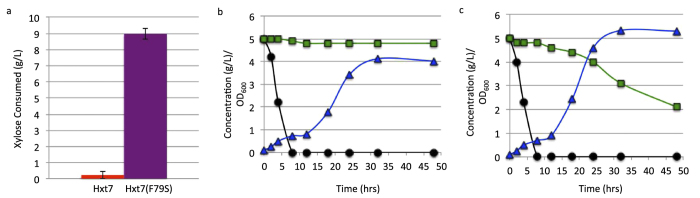
*HXT7(F79S)* is responsible for xylose uptake and for partial co-utilization of a glucose/ xylose mix. (**a**) Strains expressing only low-copy *HXT7* (JBEI-9010) or *HXT7(F79S)* (JBEI-9009) were grown at 30 °C, and the amount of xylose consumed from YP, 2% xylose medium was examined after 48 hours. The mean of three replicates is shown with standard deviation error bars. Colors correspond to representative growth curves from Fig. 3. (b) Growth and consumption of 0.5% glucose, 0.5% xylose at 30 °C in strains expressing wild type *HXT7* (JBEI-9010), or (c) *HXT7(F79S)* (JBEI-9009). The residual glucose (black circles) and xylose (green squares) are plotted for each time-point in g/L, as is the OD_600_ (blue triangles). Each chart is the mean of three biological replicates.

To verify that the growth seen in the *HXT7(F79S)* strains were indeed due to increased xylose uptake, the amounts of xylose consumed from YP, 2% xylose medium were examined after 48 hours. High-performance liquid chromatography (HPLC) analysis established that strains expressing wild-type *HXT7* only consumed 0.2 ± 0.2 g/L xylose, while strains expressing the mutant *HXT7(F79S)* consumed 9.0 ± 0.3 g/L (Fig. 4a), corroborating that the growth seen in *HXT7(F79S)* expressing strains is due to increased xylose uptake.

To examine the co-utilization of glucose and xylose, strains were grown in 0.5% glucose, 0.5% xylose medium, inoculated at a starting OD_600_ of 0.1, and monitored periodically for sugar consumption and OD_600_. Strains harboring the wild type and mutant Hxt7 transporters showed similar, rapid consumption of glucose. However, the wild type *HXT7* strain did not consume any xylose during the 48-hour experiment (Fig. 4b), while the *HXT7(F79S)* mutant steadily consumed 3 g/L of xylose during the time-course and attained a higher final OD_600_ (Fig. 4c). The strain harboring the *HXT7(F79S)* mutation displayed a substantial improvement in glucose and xylose consumption, demonstrating that the Hxt7(F79S) transporter enables partial co-utilization of glucose and xylose in a mixed-sugar source.

Recently, Farwick *et al*. described a mutation in the Hxt7 transporter, N370S, that reduced glucose repression^35^. We combined the N370S mutation with F79S to test if this would allow for a transporter that possesses both reduced glucose repression with improved xylose import kinetics. Sugar consumption and OD_600_ were monitored from strains expressing *HXT7*, *HXT7(F79S)*, *HXT7(N370S)*, or *HXT7(F79S,N370S)* in 0.5% glucose, 0.5% xylose medium (Fig. S3). All strains rapidly consumed the glucose. As before, the wild type *HXT7* strain did not consume xylose during the 48-hours (Fig. S3, panel a), while the *HXT7(F79S)* mutant consumed about 3 g/L of xylose (Fig. S3, panel b). Surprisingly, the *HXT7(N370S)* mutant did not show xylose uptake or glucose insensitivity in our minimally engineered strain background (Fig. S3, panel c), and when combined with the F79S mutation, the strain did not show glucose insensitivity, and consumed less than 1 g/L of xylose (Fig. S3, panel d).

### Kinetic measurement of Hxt7 and Hxt7(F79S) xylose transport

**Figure 5:**
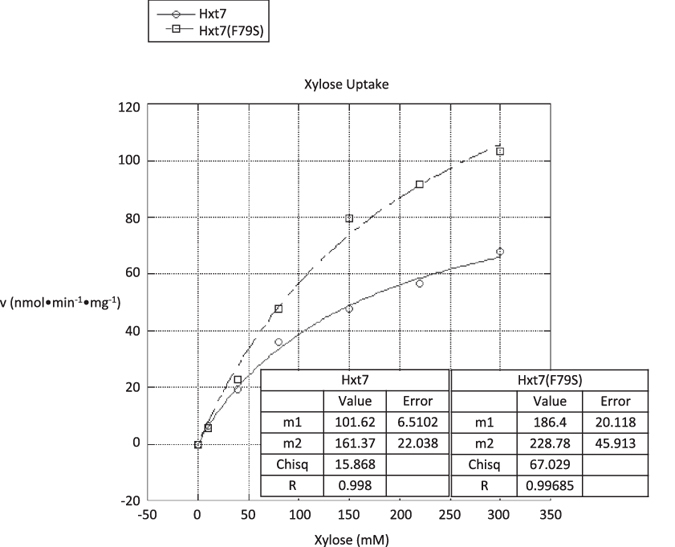
Xylose uptake kinetics of *S. cerevisiae* strains expressing *HXT7*, or *HXT7(F79S)*. Initial xylose uptake (10 s) was measured at 30 °C over a concentration range of 10 to 300 mM xylose. To arrive at Michaelis-Menten kinetic parameters, global curve fitting analysis was applied to the mean of three independent measurements at each concentration for both *HXT7* (solid line, open circle; JBEI-9012), and *HXT7(F79S)* (dashed line, open square; JBEI-9011).

In order to understand how *HXT7(F79S)* affects transport, the kinetic properties of the mutant and wild-type transporters were assayed with radioactive sugar uptake assays (Fig. 5). Strains deleted for all hexose transporters that can transport xylose (*hxt1∆, hxt2∆, hxt4∆, hxt5∆, hxt7∆, gal2∆*) were transformed with low-copy plasmids expressing either *HXT7* or *HXT7(F79S)*. The *6∆* strain was used in lieu of the well-established EBY.VW4000–complete hexose transport-deficient strain, because of the recent report that EBY.VW4000 possesses extensive chromosomal abnormalities^40^. The wild-type Hxt7 transporter was confirmed to be a low-affinity xylose transporter with a Km of 161.4 ± 22 mM, and a Vmax of 101.6 ± 6.5 nmol•min^−1^•mg^−1^ for xylose, similar to previously published values^22^,^35^. The Hxt7(F79S) mutant transporter displayed a similar xylose substrate affinity of 228.8 ± 45.9 mM, but showed about a two-fold increase in xylose transport velocity (Vmax = 186.4 ± 20.1 nmol•min^−1^•mg^−1^) over its wild-type counterpart.

## Discussion

The need to engineer a *S. cerevisiae* strain that can consume both pentose and hexose sugars, ideally together, is well recognized as important for engineering yeast to produce fuels and commodity chemicals. The main impediment to the realization of this goal is the lack of necessary xylose transporters in *S. cerevisiae*. Specifically, two aspects of xylose transport need improvement before the goal of co-utilization can be reached: (1) transport rates, (2) glucose inhibition. The latter problem has been recently addressed using a selection approach to generate glucose insensitive Gal2 and Hxt7 variants^35^. Here we generate an endogenous xylose transporter that has high rates of transport while maintaining high growth rates on xylose. This transporter, Hxt7(F79S), also allows for partial co-utilization of glucose and xylose, thereby decreasing the cultivation time needed to consume all sugars from a mixed-sugar source.

In our efforts we compiled several commonly used cytosolic xylose utilization genes and genetic modifications that served as our engineered strain and as the basal strain for lab evolution (Fig. 1a). A lab evolution regime, using serial dilution and plating on solid medium, and 2% xylose as the sole carbon source led to the appearance of colonies that could sustain significant growth on xylose (Fig. 1b). The phenotype was tracked to a single mutation in the Hxt7 protein. The *HXT7(F79S)* mutation allows for an improvement in xylose transport rates (Vmax), as well as provides for growth on xylose, and partial glucose/xylose co-utilization.

The mutant residue F79 lies within a previously reported G-G/F-XXXG motif located from amino acids 75 to 80 (Fig. S4), although in their report Young *et al*.^34^ incorrectly report the locus of the Hxt7 GGFVFG motif as amino acids 36 to 41. Our discovery further highlights the importance of this region, not just for rewiring of glucose transporters for xylose as shown by Young *et al*. but also for increasing xylose transport while maintaining glucose transport capabilities. Additionally, a recent report also identifies amino acid F79 of the heterologous transporter Mgt05196p from *Meyerozyma guilliermondii* as one of several amino acids that show slight improvement of growth on glucose and xylose when mutated to alanine^41^.

Combining the previously reported N370S mutation with the F79S mutation did not result in a xylose transporter with both reduced glucose repression and increased xylose transport (Fig. S3). This is likely due to the previously identified N370S mutation not providing for glucose repression in our strain which differs significantly from the EBY.VW4000 background used in Farwick *et al*. that contains known extensive chromosomal abnormalities^40^ that may be required for the reported phenotype. The Hxt7N370S phenotype may also require the protein to be overexpressed^35^, unlike the physiological levels used in all of our experiments with single, low-copy plasmid with native promoter. The reduced xylose uptake/utilization of the double mutant *HXT7(F79S,N370S)* over the single mutant *HXT7(F79S)* could also be due to epistasis or protein unfolding.

Lab evolution of *S. cerevisiae* is another commonly used strategy to obtain variants that have improved xylose utilization phenotypes. Several such studies are reported in the literature and each has resulted in the identification of key metabolic and regulatory genes^42^,^43^,^44^,^45^,^46^. Our study is the first lab evolution to find a mutation in a plasma membrane sugar transporter (*HXT7*), highlighting the importance of selecting appropriate starting strains and selective pressures to obtain desired phenotypes. While evolutionary selection is a powerful approach, it cannot sample all possible mutations in the amount of time given in the lab. Directed evolution approaches have produced heterologous and hybrid transporters with improved kinetics, such as the *Candida intermedia* Gxs1 pump, the *S. stipitis* Xut3 transporter, and the chimeric *S. cerevisiae* Hxt36 protein^47^,^48^. Recent directed evolution of *HXT7* provided promising results^34^,^35^, revealing that more saturated attempts may be a good next step for further *HXT7* engineering.

Native *S. cerevisiae* sugar transporters all have much greater specificity and uptake rates for hexose sugars. Several of the native hexose transporters can leak in xylose, and the one with the best xylose specificity, Hxt7, displays a low Km of 161 mM. Hxt7 also exhibits a meager uptake rate of 101 nmol•min^−1^•mg^−1^, does not alone support growth on xylose, and is inhibited by the presence of other sugars^22^. Some heterologous xylose-transporters have been identified, and have helped improve xylose utilization^31^. However, their performance has been hampered by poor growth rates, low substrate affinities, transport rates, or substrate inhibition. Recently success in engineering of native transporters has resulted in the identification of a xylose transport sequence motif^34^, and the generation of glucose insensitive strains^35^. These approaches also resulted in diminished uptake rates (Vmax), and resulted in modest growth on xylose, which are of limited value to future mixed sugar co-utilization. The *HXT7(F79S)* mutation alone enhanced the xylose transport rate (Vmax), which enables growth on xylose in a minimally engineered background strain. The mutation decreases doubling times from over 150 hours to nine hours (Fig. 1b), and doubles xylose transport rates to 186.4 nmol•min^−1^•mg^−1^ (Fig. 5), without affecting xylose affinity.

Using the structure of the bacterial homolog of the yeast Hxt proteins, XylE^38^, we were able to model the structure of Hxt7 (Fig. 2b), and to address possible mechanisms of action for Hxt7(F79S). The model predicts that the mutated residue, F79, faces inward towards the central sugar-binding pore, similarly to the residues previously identified as critical to glucose binding^49^. The mutated Phe residue of Hxt7 aligns with a Phe residue that participates in xylose binding for XylE, providing support for the importance of this residue in Hxt7 sugar transport. The amino acid substitution from a Phe to a Ser shifts the Hxt7 sugar-transporting pore towards polarity. This perhaps provides for increased xylose transport rates by allowing for additional hydrogen bonding between Ser and xylose; by allowing for additional water molecules to enter, thereby contributing to substrate binding through water-mediated hydrogen binding; or by allowing for a conformational change that favors xylose transport. Because we do not observe an increase in xylose affinity (Km) with Hxt7(F79S), the latter two mechanisms are more likely. Further structural information for the yeast Hxt proteins will enhance our understanding of xylose transport, and help to solidify the exact mechanism of how the *HXT7(F79S)* mutation affects xylose transport.

Both of the coding mutations found in the evolved strain were reasonable candidates for impacting sugar utilization. The causal mutation in *HXT7* was not surprising since the native transporter had been previously shown to provide for the highest intracellular accumulation of xylose in *S. cerevisiae*^26^. The only other mutation in our xylose-evolved strain, *YDL176W(D504H)*, had an almost indiscernible impact on this phenotype by itself (Fig. S2). Although *YDL176W* is largely uncharacterized, it is predicted to be involved in fructose-1,6-bisphosphatase (Fbp1) degradation and a member of the glucose-induced degradation (GID) complex^50^,^51^,^52^, making it a likely target for affecting sugar utilization. When *S. cerevisiae* are starved of glucose for prolonged periods of time, gluconeogenic enzymes such as Fbp1 are induced^53^. Therefore, one possible explanation for this mutation is that it resulted not from the adaptation to xylose, but instead from long-term glucose starvation. Alternatively, components of the GID complex have been implicated in degradation of Hxt7^54^. Perhaps Ydl176W(D504H) could be altering the degradation of Hxt7, explaining the slight growth improvement seen at 60 hours. However, from our studies, *HXT7(F79S)* provides the phenotype seen in the evolved strain.

Our discovery has enabled us to contribute to the list of yeast xylose utilization discoveries made to date. This invention has very broad applicability. All industries and research ventures that use *S. cerevisiae* yeast microbial hosts as their platform to convert sugar to a desired product may find this mutant transporter useful. Moreover, the xylose utilization phenotype reported here is due to expression of a single nucleotide substitution in a single copy of *HXT7*, making this discovery easily transferable to established industrial strains. The *HXT7(F79S)* mutation allows yeast to better use xylose, thus allowing it to use the main sugars (glucose and xylose) present in the mixes that arise from saccharification of plant biomass. This ability would be desirable specifically to industries and ventures that are manufacturing bulk compounds and chemicals and that wish to have inexpensive and sustainable biomass as the feedstock.

With this discovery, both aspects needed for xylose transport have been engineered independently. The future of xylose utilization will need to focus on combining these properties into one transporter. Incorporating motif modifications to reduce glucose repression and to improve import, in addition to other genetic modifications that also improve xylose utilization may result in the true elimination of diauxic growth that is typical in mixed carbon sources.

## Methods

### Strains and media

A complete list of strains and plasmids used in this study can be found in Tables S1 and S2 (see Additional File 1), and are available through the JBEI registry (http://public-registry.jbei.org^55^). Yeast cells were grown in standard rich (YP, yeast extract-peptone) or synthetic defined media (SD, yeast nitrogen base with CSM amino acids (Sunrise Science Products) for plasmid selection) with 2% sugar, unless otherwise stated. For yeast kanamycin resistance selection, 250 μg/ml of geneticin (G418) was used in rich medium. Bacteria were grown in LB with 50 μg/ml carbenicillin.

*S. cerevisiae* strains were transformed with plasmids using the conventional lithium acetate method^56^. DNA cloning was performed using standard techniques; T4 DNA polymerase-mediated (Fermentas) ligations or Gibson assembly in *Escherichia coli*, or homologous recombination in *S. cerevisiae*. Plasmids were recovered from S*. cerevisiae* by lysing the cells mechanically with glass beads, followed by plasmid mini-prep (Qiagen). Chromosomal gene deletions were generated by integration of PCR products flanked by *loxP* sites^57^.

### Strain evolution

A BY4742 *gre3∆* strain expressing *Piromyces sp. XI* (Pi-xylA), and *XKS1* from two high-copy plasmids was evolved in SD –URA –HIS, with 2% xylose. The 4 mL culture was maintained at 30 °C, shaking at 200 revolutions/min. Mutants with increased specific growth rates were selected through dilution of the culture when turbidity was seen. At periodic intervals, the culture(s) were plated onto solid SD –URA –HIS, 2% xylose medium, and several of the fastest-growing colonies were selected for independent evolution in liquid culture. This process was repeated, selecting for the fastest growing isolates at each round, until culture saturation was achieved within one to two days of dilution. In total, the evolution process took approximately three months until satisfactory growth was achieved. At the end of the process, about one dozen clones were re-streaked and tested individually for xylose growth. One of the best performing clones, 7a2c (JBEI_ScMO002), was selected and prepared for genome sequencing.

### Genome sequencing

Five μg of total gDNA was extracted from the parental and evolved strains, and sent to the Department of Energy Joint Genome Institute (DOE JGI, Walnut creek) for whole genome resequencing. Resequencing data associated with this study can be found via NCBI SRA accession numbers SRX298977 and SRX298863. Burrows-Wheeler Aligner (BWA) was used to align reads, and Bcftools to assign SNPs and indels. Sequencing files were analyzed using Integrated Genome Viewer software^58^.

### Xylose growth experiments

Strains were grown overnight in SD –LEU –URA, 1.4% glucose, 0.6% xylose medium. Cells were pelleted and resuspended to a final OD_600_ of 0.1 in 1 mL of SD –LEU –URA, 2% xylose medium in a 24-well plate. The plate was then placed into the BioTek Synergy 4, preheated to 30 °C, and the growth was monitored by taking the OD_600_ every fifteen minutes, for 60 hours.

For experiments associated with Fig. 4, strains were grown overnight in SD –LEU –URA, 1.4% glucose, 0.6% xylose medium. Cells were pelleted and resuspended to a final OD_600_ of 0.1 in 5 mLs of YP, 2% xylose medium (Fig. 4a), or YP, 0.5% glucose, 0.5% xylose (Fig. 4b,c) in standard culture tubes at 30 °C. Samples were periodically taken and monitored for sugar concentrations and OD_600_ for 48 hours.

### Analysis of glucose and xylose concentrations

The concentrations of sugars were quantified on an Agilent Technologies 1200 series HPLC equipped with an Aminex H column. Samples were filtered through 0.45 μm VWR filters to remove cells, and 5 μl of each sample was injected onto the column, preheated to 50 °C. The column was eluted with 4 mM H_2_SO_4_ at a flow rate of 600 μl/min for 25 minutes. Sugars were monitored by refractive index detector, and concentrations were calculated by comparison of peak areas to known standards.

### Radioactive sugar uptake

Uptake of ^14^C-xylose was used to determine the Michaelis–Menten parameters for Hxt7(F79S). 1-^14^C-xylose was purchased from American Radiolabeled Chemicals. Twelve mL overnight cultures grown in SD –URA, 1.4% glucose, 0.6% xylose medium, were diluted to an OD_600_ of 0.1/ml in 50 mL of medium and allowed to grow until mid-log phase (OD_600_ 0.5 to 0.8). Twenty ODs of cells were centrifuged at 3000 x g for 5 minutes and washed once with 10 mL of 0.1 M potassium phosphate buffer, pH 6.8. Cultures were then resuspended in 300 μl of 0.1 M potassium phosphate buffer, pH 6.8, and warmed to 30 °C. Twenty-five μl of cells were then mixed with an equal amount of radiolabeled sugar solutions, producing final mixed sugar concentrations between 10 mM and 400 mM. Ten seconds after mixing, the samples were filtered through 0.2 μm Whatman Nuclepore filters (GE Healthcare), and washed with 10 mL ice-cold 0.1 M potassium phosphate, 500 mM xylose buffer. Filters were subsequently placed in 4 mL Ecoscint XR scintillation fluid (National Diagnostics) and counted in a LS 6500 scintillation counter (Beckman-Coulter). KaleidaGraph software (Synergy Software) was used to plot the data, and to arrive at Michaelis–Menten kinetic parameters for each transporter. All assays were performed in biological triplicate. One outlier with accelerated uptake was discarded from the 300 mM *HXT7(F79S)* data set.

### Protein Structure Prediction

The predicted Hxt7 structure (Fig. 2b) was generated using Phyre^37^, and the published XylE structures (PDB: 4GBY and 4GBZ). Three-dimensional, structural images were created with PyMOL (Schrödinger, LLC.).

## Additional Information

**How to cite this article**: Reider Apel, A. *et al*. Evolved hexose transporter enhances xylose uptake and glucose/xylose co-utilization in *Saccharomyces cerevisiae*. *Sci. Rep*. **6**, 19512; doi: 10.1038/srep19512 (2016).

## Supplementary Material

Supplementary Information
