# Supplementary material for: Evolved hexose transporter enhances xylose uptake and glucose/xylose co-utilization in Saccharomyces cerevisiae
Source: Sci Rep. 2016 Jan 19;6:19512. doi: 10.1038/srep19512 (PMC4726032; doi:10.1038/srep19512)
Supplement: Supplementary Information [file srep19512-s1.pdf]

Supplementary Information

Evolved hexose transporter enhances xylose uptake and glucose/xylose co-utilization in *Saccharomyces cerevisiae*

Amanda Reider Apel[1,2], Mario Ouellet[1,2], Heather Szmidt-Middleton[1,2], Jay D Keasling[1,2,3,4], Aindrila Mukhopadhyay[1,2*]

[1]Joint BioEnergy Institute, 5885 Hollis St, Emeryville, CA 94608, USA

[2]Biological Systems and Engineering Division, Lawrence Berkeley National Laboratory, Berkeley, CA 94720,USA

[3]Department of Bioengineering, University of California, Berkeley, CA 94720, USA

[4]Department of Chemical & Biomolecular Engineering, University of California, Berkeley, CA 94720, USA

*Corresponding Author

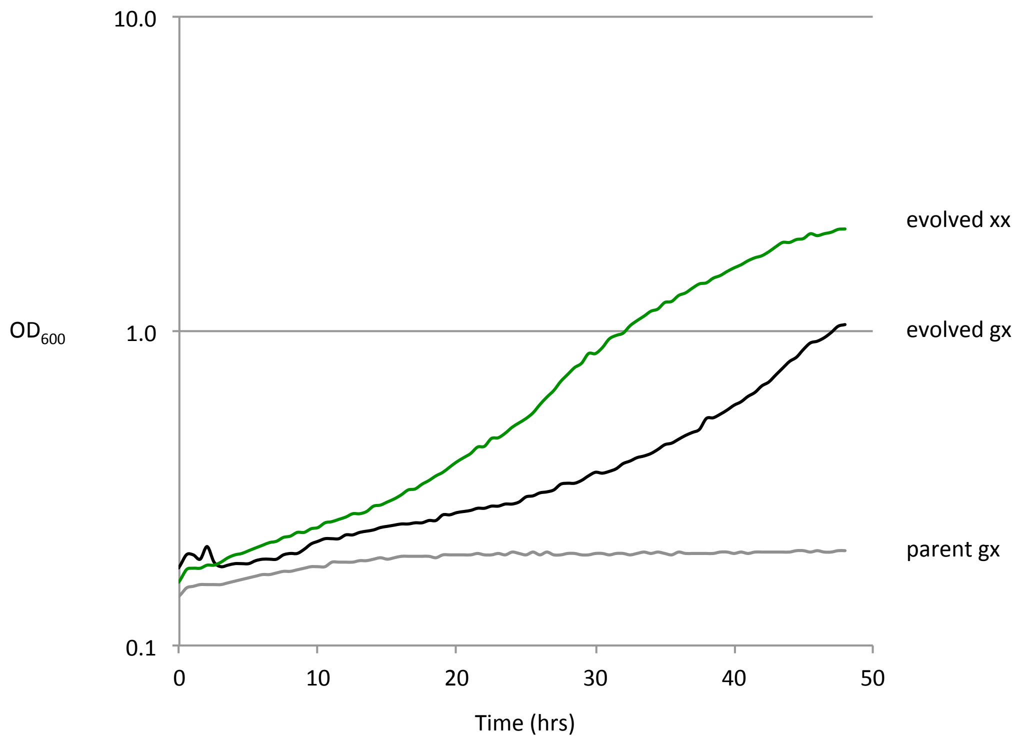

Fig. S1. Pre-growth of the evolved strain on glucose leads to a lag in growth and utilization xylose. The parent strain (JBEI_ScMO001) and evolved strain (JBEI_ScMO002) were pre-grown in SD, 2% glucose medium (g) or SD, 2% xylose medium (x), prior to culturing in SD, 2% xylose medium (x) at 30 ˚C. Once in the final xylose medium, OD_{600} was measured every 15 minutes for 48 hours. The experiment was conducted in triplicate, and the representative curves are shown. Y-axis is shown in log base 10 scale.

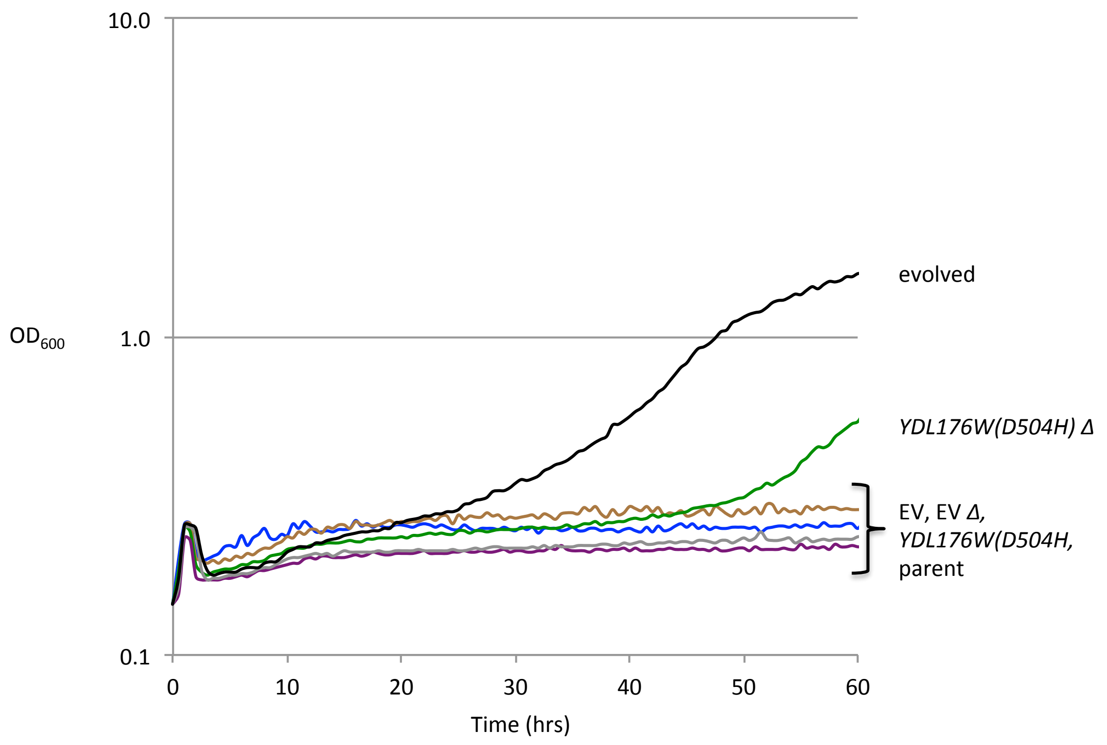

Fig. S2. *YDL176W(D504H)* does not contribute significantly to growth on xylose. *YDL176W(D504H)* or empty vector (EV) were expressed in strains wild-type (JBEI-9013 EV; JBEI-9014 *YDL176W(D504H)*) or deleted (Δ) for *ydl176w* (JBEI-9015 EV; JBEI-9016 *YDL176W(D504H)*). The strains were grown in SD, 2% xylose medium at 30 ˚C, and the OD_{600} was measured every 15 minutes for 60 hours. The experiment was conducted in triplicate, and the representative curves are shown. For *YDL176W(D504H)* (green), only two of the three clones showed growth. Y- axis is shown in log base 10 scale.

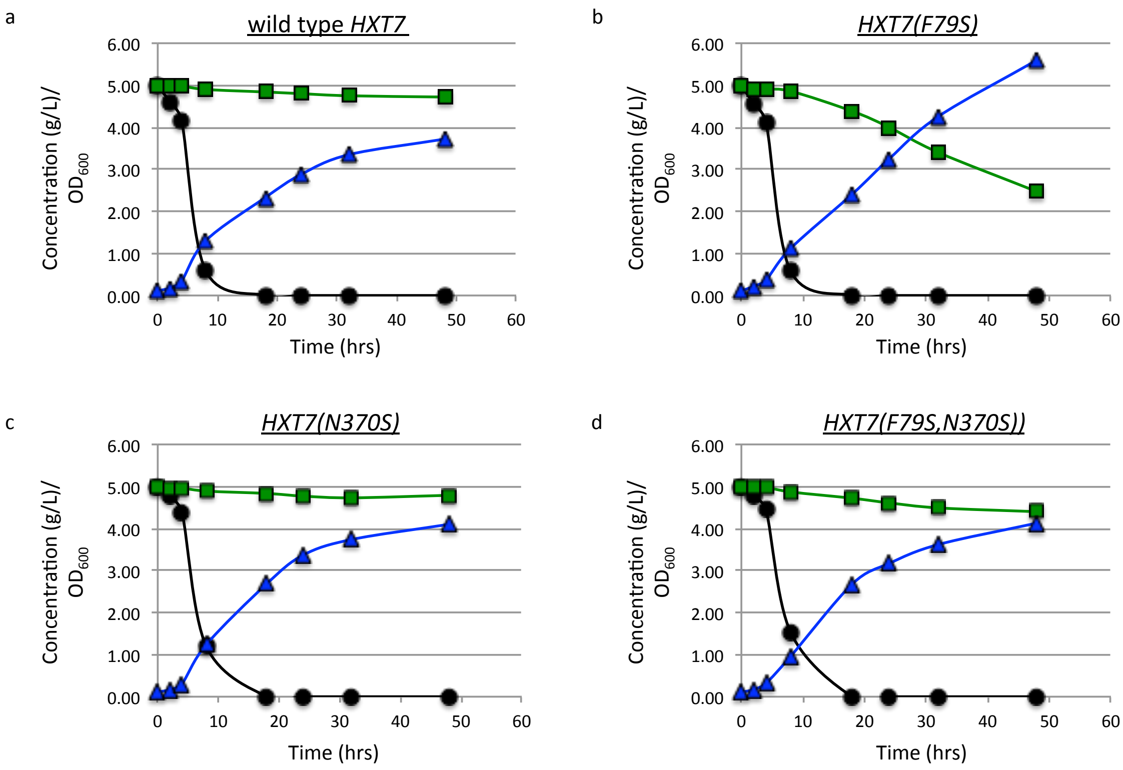

Fig. S3. Combining the *F79S and N370S* mutations in Hxt7 does not provide synergistic glucose insensitivity and partial glucose/ xylose co-utilization phenotype. Growth and consumption of 0.5% glucose, 0.5% xylose at 30 ˚C in strains expressing (a) wild type *HXT7* (JBEI-9010), (b) *HXT7(F79S)* (JBEI-9009), (c) *HXT7(N370S)* (JBEI-13185), or (d) *HXT7(F79S,N370S)* (JBEI-13186). The residual glucose (black circles) and xylose (green squares) are plotted for each time-point in g/L, as is the OD_{600} (blue triangles). Each chart is the mean of three biological replicates.

1 MSQDAAIAEQ TPVEHLSAVD SASHSVLSTP SNKAERDEIK AYGEGEEHEP 50

51 VVEIPKRPAS AYVTVSIMCI MIAFGGFV**F**G WDTGTISGFI NQTDFIRRFG 100

101 MKHKDGTNYL SKVRTGLIVS IFNIGCAIGG IILSKLGDMY GRKVGLIVVV 150

151 VIYIIGIIIQ IASINKWYQY FIGRIISGLG VGGIAVLSPM LISEVSPKHL 200

201 RGTLVSCYQL MITAGIFLGY CTNFGTKNYS NSVQWRVPLG LCFAWALFMI 250

251 GGMTFVPESP RYLAEVGKIE EAKRSIAVSN KVAVDDPSVL AEVEAVLAGV 300

301 EAEKLAGNAS WGELFSSKTK VLQRLIMGAM IQSLQQLTGD NYFFYYGTTI 350

351 FKAVGLSDSF ETSIVLGIVN FASTFVGIYV VERYGRRTCL LWGAASMTAC 400

401 MVVYASVGVT RLWPNGQDQP SSKGAGNCMI VFACFYIFCF ATTWAPIPYV 450

451 VVSETFPLRV KSKAMSIATA ANWLWGFLIG FFTPFITGAI NFYYGYVFMG 500

501 CLVFMFFYVL LVVPETKGLT LEEVNTMWEE GVLPWKSASW VPPSRRGANY 550

551 DAEEMTHDDK PLYKRMFSTK

Fig. S4. Hxt7 wild type amino acid sequence. The G-G/F-XXXG motif (from the Farwick study) is located from amino acids 75 to 80 (underlined), and the mutated residue from this study, F79, is bolded.

Table S1. *S. cerevisiae* strains used in this work

Name	Genotype	Source
JBEI_ScMO001	BY4742*; gre3Δ* pRS426.*XKS1* pRS423.*XI*	This study
JBEI_ScMO002	BY4742; *gre3Δ* pRS426.*XKS1* pRS423.*XI* xylose evolved	This study
JBEI-9005	BY4742; *gre3Δ* pMOXYL3 pRS416	This study
JBEI-9006	BY4742*; gre3Δ* pMOXYL3 pRS416.*HXT7(F79S)*	This study
JBEI-9007	BY4742*; gre3Δ* pMOXYL3 pRS416.*HXT7*	This study
JBEI-9008	BY4742*; gre3Δ hxt7Δ::loxpKanMX* pMOXYL3 pRS416	This study
JBEI-9009	BY4742*; gre3Δ hxt7Δ::loxpKanMX* pMOXYL3 pRS416.*HXT7(F79S)*	This study
JBEI-9010	BY4742*; gre3Δ hxt7Δ::loxpKanMX* pMOXYL3 pRS416.*HXT7*	This study
JBEI-9011	BY4742*; hxt1Δ::loxp hxt2Δ::loxpLEU2 hxt4ΔKanMX hxt5Δ::loxp hxt7Δ::loxp gal2Δ::loxp* pRS416.*HXT7(F79S)*	This study
JBEI-9012	BY4742*; hxt1Δ::loxp hxt2Δ::loxpLEU2 hxt4ΔKanMX hxt5Δ::loxp hxt7Δ::loxp gal2Δ::loxp* pRS416.*HXT7*	This study
JBEI-9013	BY4742*; gre3Δ*; pMOXYL3 pRS413	This study
JBEI-9014	BY4742*; gre3Δ* pMOXYL3 pRS413.*YDL176W(D504H)*	This study
JBEI-9015	BY4742*; gre3Δ ydl176wΔloxpKanMX* pMOXYL3 pRS413	This study
JBEI-9016	BY4742*; gre3Δ ydl176wΔloxpKanMX* pMOXYL3 pRS413.*YDL176W(D504H)*	This study
JBEI-13185	BY4742*; gre3Δ hxt7Δ::loxpKanMX* pMOXYL3 pRS416.*HXT7(N370S)*	This study
JBEI-13186	BY4742*; gre3Δ hxt7Δ::loxpKanMX* pMOXYL3 pRS416.*HXT7(F79S,N370S)*	This study

Table S2. Description of plasmids used in this work

Name	Description	Source
pRS426.*XKS1*	High copy, *URA3* plasmid, expressing *XKS1* under control of a *TDH3p* and a *CYC1t*	This study
pRS423.*XI*	High copy, *HIS3* plasmid, expressing yeast codon optimized, *piromyces* species *XI* under control of a *TDH3p* and a *CYC1t*	This study
pMOXL3	High copy, Leu2d plasmid, expressing *TAL1* under control of a *TEF1p* and a *ADH1t*, and *XKS1* and *piromyces* species *XI*, separately under control of a *TDH3p* and a *CYC1t*	This study
pRS416	Empty, low copy, *URA3* plasmid	
pRS413	Empty, low copy, *HIS3* plasmid	
pRS416.*HXT7(F79S)*	pRS416, expressing *HXT7(F79S)* under control of 500bp of its native promoter and terminator	This study
pRS416.*HXT7*	pRS416, expressing *HXT7* under control of 500bp of its native promoter and terminator	This study
pRS413.*YDL176W(D504H)*	pRS413, expressing *YDL176W(D504H)* under control of 500bp of its native promoter and terminator	This study
